# Comparison of the efficacy of Oxford unicondylar replacement for the treatment of spontaneous osteonecrosis of the knee versus medial knee osteoarthritis: a meta-analysis

**DOI:** 10.1186/s13018-023-04519-5

**Published:** 2024-01-22

**Authors:** Pengyu Liu, Liangliang Li, Jiaju Yang, Hao Li, Yuhua Feng, Zhipeng Qin, Min Zhang

**Affiliations:** 1 Second Hospital of Shanxi Medical University, Taiyuan, 030001 Shanxi China; 2 Tianjin Medical University General Hospital, Tianjin, 300052 China

**Keywords:** Oxford unicondylar replacement, Spontaneous osteonecrosis of the knee, Osteoarthritis of the knee, Meta-analysis

## Abstract

**Objective:**

Meta-analysis of the comparative efficacy of Oxford unicompartmental knee arthroplasty (OUKA) for the treatment of spontaneous osteonecrosis of the knee (SONK) and medial knee osteoarthritis (MKOA).

**Methods:**

A computerized search was conducted for literature related to OUKA treatments of SONK and MKOA across various databases, including the China National Knowledge Infrastructure, WAN FANG, VIP, SinoMed, Cochrane Library, PubMed, Embase, and Web of Science, covering the period from each database's inception to September 2023. Literature screening, quality assessment and data extraction were performed according to the inclusion and exclusion criteria. After extracting the literature data, RevMan 5.4 software was applied to analyse the postoperative knee function score, postoperative knee mobility, postoperative pain, bearing dislocation rate, aseptic loosening, postoperative progression of posterolateral arthritis, and revision rate.

**Result:**

A total of 9 studies were included, including 6 cohort studies and 3 matched case‒control studies. A total of 1544 knees were included, including 183 in the SONK group and 1361 in the MKOA group. The meta-analysis results showed that the SONK and MKOA groups showed a significant difference in postoperative knee function scores [MD = 0.16, 95% CI (− 1.20, 1.51), *P *= 0.82], postoperative knee mobility [MD = − 0.05, 95% CI (− 1.99. 1.89), *P *= 0.96], postoperative pain [OR = 0.89, 95% CI (0.23, 3.45), *P *= 0.87], rate of bearing dislocation [OR = 1.28, 95% CI (0.34, 4.81), *P *= 0.71], aseptic loosening [OR = 2.22, 95% CI (0.56, 8.82), *P = *0.26], postoperative posterolateral arthritis progression [OR = 2.14, 95% CI (0.47, 9.86), *P* = 0.33], and revision rate [OR = 1.28, 95% CI (0.53, 3.04), *P *= 0.58] were not statistically significant.

**Conclusion:**

OUKA treatment with SONK and MKOA can achieve similar satisfactory clinical results.

## Introduction

Spontaneous osteonecrosis of the knee (SONK) is a localised, spontaneous disease of the medial condyle of the femur, first reported by Ahlback in 1968 [1], which is commonly seen in middle-aged and elderly patients. It is distinguished from osteoarthritis by the presence of resting and nocturnal pain [2–5], though its etiology remains unknown, and excessive contact stress associated with cartilage and meniscus injuries is thought to be one of the main causes of this condition [6, 7]. Surgical treatment is usually needed for patients with severe pain, especially nocturnal pain, and Oxford unicompartmental knee arthroplasty (OUKA) has been shown to be effective in the treatment of medial knee osteoarthritis (MKOA) [8, 9]. SONK is a typical unicompartmental disease with a site of onset similar to that of MKOA, occurring mostly in the medial femoral weight-bearing area of the medial femoral condyle [10]. Some literature has shown that OUKA can achieve equal efficacy in the treatment of SONK and MKOA, but the literature is a small sample size survey and lacks a large sample size analysis. YOON et al. [11] included 11 publications on the treatment of SONK and MKOA with unicompartmental knee arthroplasty (UKA) (including fixed and movable platforms) for meta-analysis, and the results showed that cemented UKA in SONK and MKOA showed similar survival and clinical outcomes. Most current UKA prostheses are predominantly active platforms; however, meta-analyses comparing the efficacy of the two, specifically for active platform UKA, have not been reported. Therefore, the present study builds on this by further incorporating recent literature on UKA for SONK and MKOA and by limiting the UKA prosthesis type to OUKA, with the aim of more comprehensively and accurately validating its efficacy and providing more evidence-based medical evidence for clinically active platform unicompartmental knee arthroplasty.

## Materials and methods

### Literature search

The search strategy for this study was conducted in accordance with the guidelines established by the Preferred Reporting Items for Systematic Reviews and Meta-Analyses (PRISMA) guidelines [12]. The protocol was registered in the PROSPERO database (CRD42023455948). Computer searches were performed on the China National Knowledge Infrastructure, WAN FANG, VIP, SinoMed, Cochrane Library, PubMed, Embase, and Web of Science databases. The search date was from the establishment of the library to September 2023. For example, in PubMed, the search terms were "Osteonecrosis", "Avascular Necrosis of Bone", "Arthroplasties, Replacement, Knee", "Unicompartmental Knee Arthroplasty", "Partial Knee Arthroplasty" and other related terms.

### Inclusion and exclusion criteria

Inclusion criteria: (1) Study population: patients with medial compartment OA of the knee or SONK of the medial femoral condyle, preoperative examination suggesting good anterior and posterior cruciate ligaments as well as medial and lateral collateral ligaments, and no sex or age limitations; (2) intervention: OUKA; (3) outcome indices: postoperative knee function scores, postoperative knee mobility, postoperative pain, rate of bearing dislocation, aseptic looseness, postoperative posterolateral arthritis progression, and revision rate. Exclusion criteria: (1) surgery other than medial UKA; (2) number of knees without separate reporting of osteonecrosis and osteoarthritis; (3) implant survival or clinical outcomes without separate reporting of osteonecrosis and osteoarthritis; and (4) duplicates of publications, animal studies, reviews or systematic evaluations, case reports, unofficial publications, commentaries, conference abstracts, and research literature with incomplete data. (5) Follow-up period of less than 1 year.

### Literature screening and data extraction

Literature screening and data extraction were carried out independently by two researchers based on inclusion and exclusion criteria, and the extracted information was put into a uniform Excel sheet and then cross-checked. Any disagreement was resolved by discussion or decided by a third senior author after intervention and discussion. Extracted data included authors' names, date of publication, type of study, type of prosthesis, basic characteristics of the study population, and duration of follow-up.

### Literature quality assessment

Quality assessment of the included cohort and case‒control studies based on the Newcastle‒Ottawa Scale (NOS) [13] was carried out independently by two researchers, with disagreements discussed and resolved if they occurred, and with a third researcher if disagreements remained.

### Statistical analysis

Meta-analysis was conducted using RevMan 5.4 software provided by the Cochrane Collaboration. The 95% confidence interval (CI) was calculated, the mean difference (MD) was used as the effect size for continuous variables, odds ratio (OR) was used as the effect size for dichotomous variables, the heterogeneity between studies was assessed by the chi-square test and the I^2^, and the fixed-effect model was used when (*P *> 0.05, I^2^ ≤ 50%) suggested that when (*P *> 0.05, I^2^ ≤ 50%) suggests less heterogeneity between studies, then fixed effects model analysis was used; if (*P *≤ 0.05, I^2^ > 50%) suggests greater heterogeneity between studies, then random effects model analysis was used. *P *≤ 0.05 was considered a statistically significant difference.

## Results

### Literature search results

**Fig. 1:**
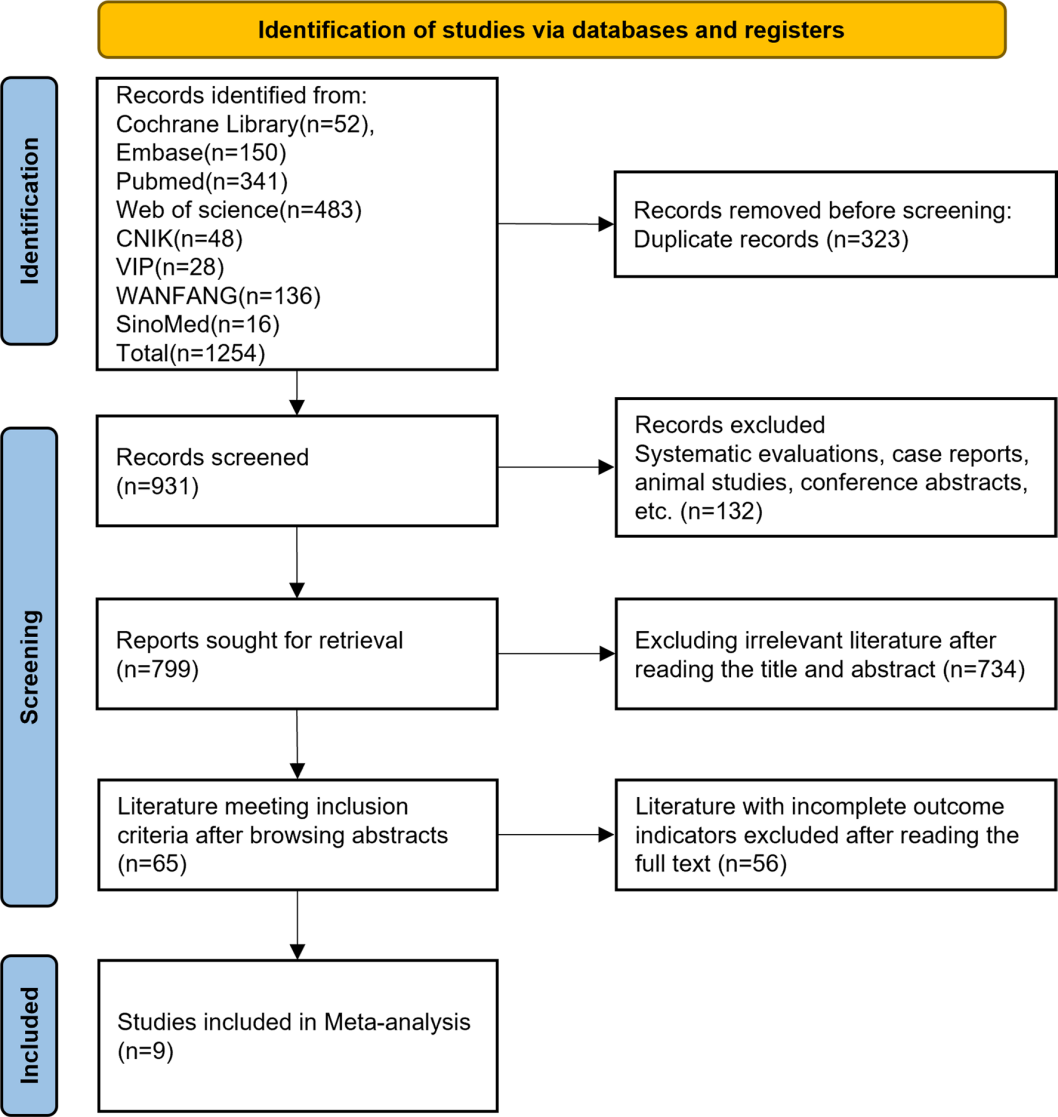
Flowchart of the search process of our study

Based on the search strategy, a total of 1254 relevant studies were retrieved. A total of 931 articles remained after excluding duplicates, 799 articles remained after excluding reviews or systematic evaluations, case reports, animal studies, unofficial publications, reviews, conference abstracts and incomplete data, 65 articles remained after excluding irrelevant articles after reading the titles and abstracts, and 9 articles were finally included after excluding incomplete outcome indicators after reading the full text [14–22], with a total of 1544 knees, including 183 cases in the SONK group and 1361 cases in the MKOA group. The literature screening process is shown in Fig. 1.

### Basic characteristics and quality assessment of the study

A total of six retrospective cohort studies [14, 16, 18–20, 22] and three matched case‒control studies [15, 17, 21] were included, of which Xue et al. [19] only provided 5-year cumulative survival rates for UKA in SONK and MKOA (98.7% and 98.8%, respectively, *p *> 0.05), and there was no statistical significance in the survival rates for either. The quality of nine studies was evaluated using the NOS rating scale out of 9. Two [15, 17] of the included studies scored 8, and seven [14, 16, 18–22] scored 7, which were all of high quality. The general information and quality evaluation of the included literature are shown in Table 1.

**Table 1:** Basic characteristics and quality assessment of the included studies

ID	Study design	Implant used	Number of cases			Sex (M/F)		Age		BMI		Mean follow-up		Effective outcome indicators	Quality rating
			SONK	MKOA	Total	SONK	MKOA	SONK	MKOA	SONK	MKOA	SONK	MKOA		
Yang [14]	Retrospective cohort study	IV	15 (15)	65 (72)	80 (87)	0/15	25/47	66.8 ± 6.1	64.0 ± 7.6	26.4 ± 4.8	26.0 ± 3.7	2.7 ± 1.4	3.0 ± 1.3	④⑤⑥⑦	7
Zhao [15]	Matched case–control study	IV	20 (20)	20 (20)	40 (40)	4/16	4/16	71.9 ± 8.2	72.5 ± 8 2	NA		2.0 ± 1.5(1–2.7)		①②③④⑤⑥	8
Ji [16]	Retrospective cohort study	IV	7	239	245 (246)	37/208		64.3 (50–76)		NA		2.8 (1–8)		③④⑤⑥⑦	7
Zhang [17]	Matched case–control study	III	29	29	58	12/17	12/17	63.83 ± 9.23	65.69 ± 11.07	25.44 ± 3.48	24.21 ± 3.71	3.7 ± 1.2	3.7 ± 1.2	②③④⑤⑥⑦	8
Langdown [21]	Matched case–control study	III	27 (29)	26 (28)	53 (57)	23/29	22/28	73 (43–88)	71 (46–85)	NA		5.2(1–13)	4.8(1–13)	①③④⑤⑥	7
Zermatten [18]	Retrospective cohort study	II	10 (10)	32 (38)	42 (48)	11/37		69.2 (57–82)		NA		NA (1–11)		④⑤⑥⑦	7
Heller [22]	Retrospective cohort study	III	9	33	42 (42)	11/31		63 (45–80)		NA		2.7 (2–5)		③④⑤⑦	7
Ma [20]	Retrospective cohort study	III	23	235	258	7/16	74/161	71.6 ± 8.53	71.0 ± 8.14	25.02 ± 2.03	25.23 ± 1.85	5(2–9)		①④⑤⑥⑦	7
Xue [19]	Retrospective cohort study	III	41	667	708	295/339		67.8 ± 10.2		30.5 ± 1.4		5.3 (3.0–6.5)	5.3 (3.0–6.6)	NA	7

*SONK* Spontaneous osteonecrosis of the knee, *MKOA* Medial knee osteoarthritis, *NA* Not available

① postoperative knee function score, ② postoperative knee mobility, ③ postoperative pain, ④ spacer dislocation rate, ⑤ aseptic loosening, ⑥ progression of postoperative posterolateral arthritis, ⑦ renovation rate

### Meta-analysis results

#### Postoperative knee function score

**Fig. 2:**
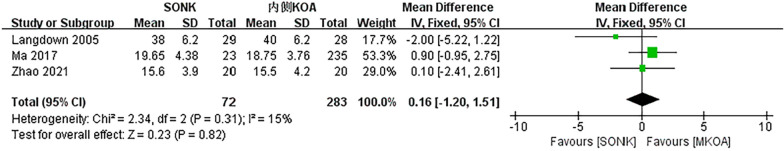
Comparison of postoperative knee function scores between the SONK group and the MKOA group

Three publications [15, 20, 21] compared posttreatment knee function scores in a total of 355 knees, 72 in the SONK group and 283 in the MKOA group. There was little heterogeneity between the findings (*P *= 0.31, I^2^ = 15%), so a fixed effects model was used. The results showed MD = 0.16, 95% CI (− 1.20, 1.51), *P *= 0.82, and the difference was not statistically significant (Fig. 2).

#### Postoperative knee mobility

**Fig. 3:**
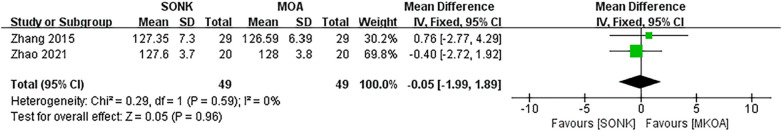
Comparison of postoperative knee mobility between the SONK and MKOA groups

Two publications [15, 17] compared knee mobility after treatment in a total of 98 knees, 49 in the SONK group and 49 in the MKOA group. There was little heterogeneity between the findings (*P *= 0.59, I^2^ = 0%), so a fixed effects model was used. The results showed MD = − 0.05, 95% CI (− 1.99, 1.89), *P *= 0.96, and the difference was not statistically significant (Fig. 3).

#### Postoperative pain

**Fig. 4:**
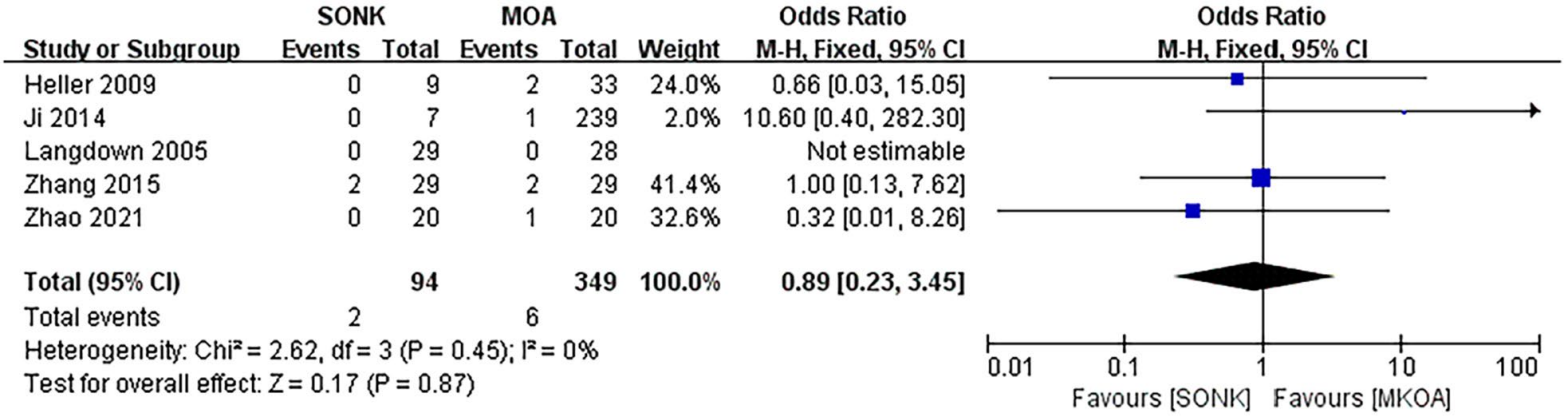
Comparison of postoperative pain between the SONK and MKOA groups

Five papers [15–17, 21, 22] compared postoperative pain at the last follow-up in a total of 443 knees, 94 in the SONK group and 349 in the MKOA group. There was little heterogeneity between the findings (*P *= 0.45, I^2^ = 0%), so a fixed effects model was used. The results showed OR = 0.89, 95% CI (0.23, 3.45), *P *= 0.87, and the difference was not statistically significant (Fig. 4).

#### Bearing dislocation rate

**Fig. 5:**
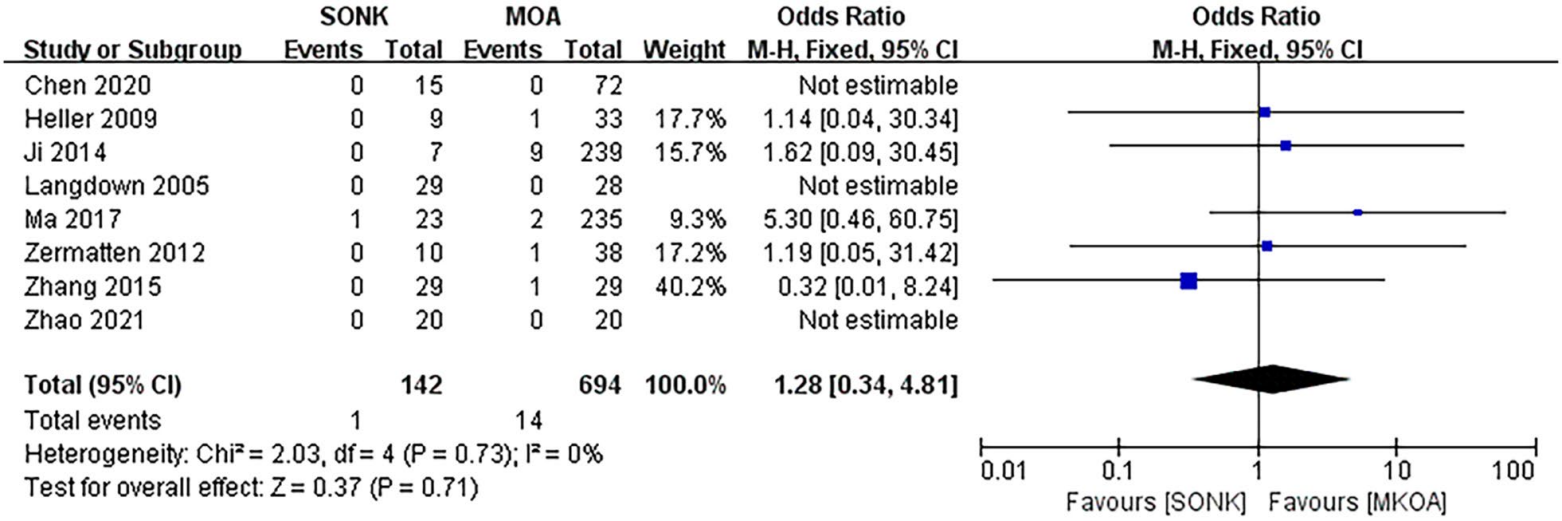
Comparison of the postoperative bearing dislocation rate between the SONK and MKOA groups

Eight papers [14–18, 20–22] compared bearing dislocation at the final follow-up in a total of 836 knees, 142 in the SONK group and 694 in the MKOA group. There was little heterogeneity between the findings (*P *= 0.73, I^2^ = 0%), so a fixed effects model was used. The results showed OR = 1.28, 95% CI (0.34, 4.81), *P *= 0.71, and the difference was not statistically significant (Fig. 5).

#### Aseptic loosening

**Fig. 6:**
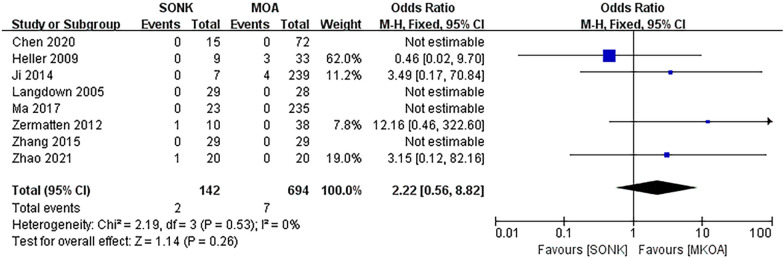
Comparison of postoperative aseptic loosening in the SONK and MKOA groups

Eight papers [14–18, 20–22] compared aseptic loosening at the final follow-up in a total of 836 knees, 142 in the SONK group and 694 in the MKOA group. There was little heterogeneity between the findings (*P *= 0.53, I^2^ = 0%), so a fixed effects model was used. The results showed OR = 2.22, 95% CI (0.56, 8.82), *P *= 0.26, and the difference was not statistically significant (Fig. 6).

#### Postoperative progression of posterolateral osteoarthritis

**Fig. 7:**
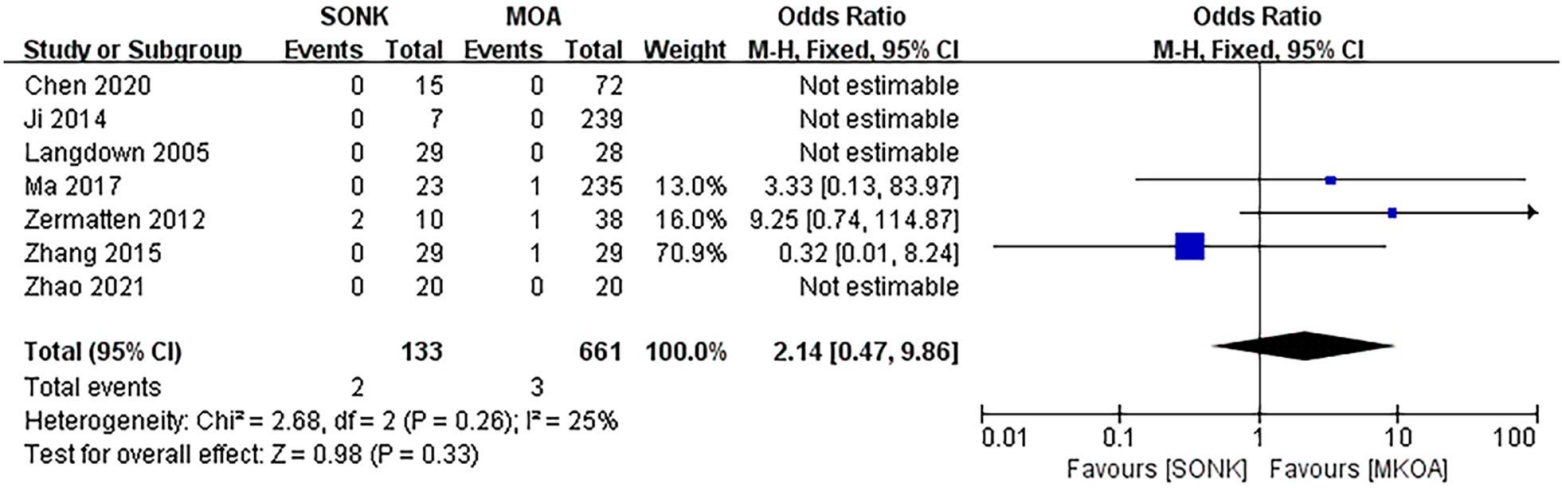
Comparison of postoperative progression of posterolateral arthritis in the SONK and MKOA groups

Seven papers [14–18, 20, 21] compared the progression of posterolateral osteoarthritis at the last follow-up in a total of 794 knees, 133 in the SONK group and 661 in the MKOA group. There was little heterogeneity between the findings (*P *= 0.26, I^2^ = 25%), so a fixed-effects model was used. After the test for heterogeneity, *P *= 0.26, I^2^ = 25%, there was no significant heterogeneity between studies, and the fixed effect model was used. The results showed OR = 2.14, 95% CI (0.47, 9.86), *P *= 0.33, and the difference was not statistically significant (Fig. 7).

#### Revision rates

**Fig. 8:**
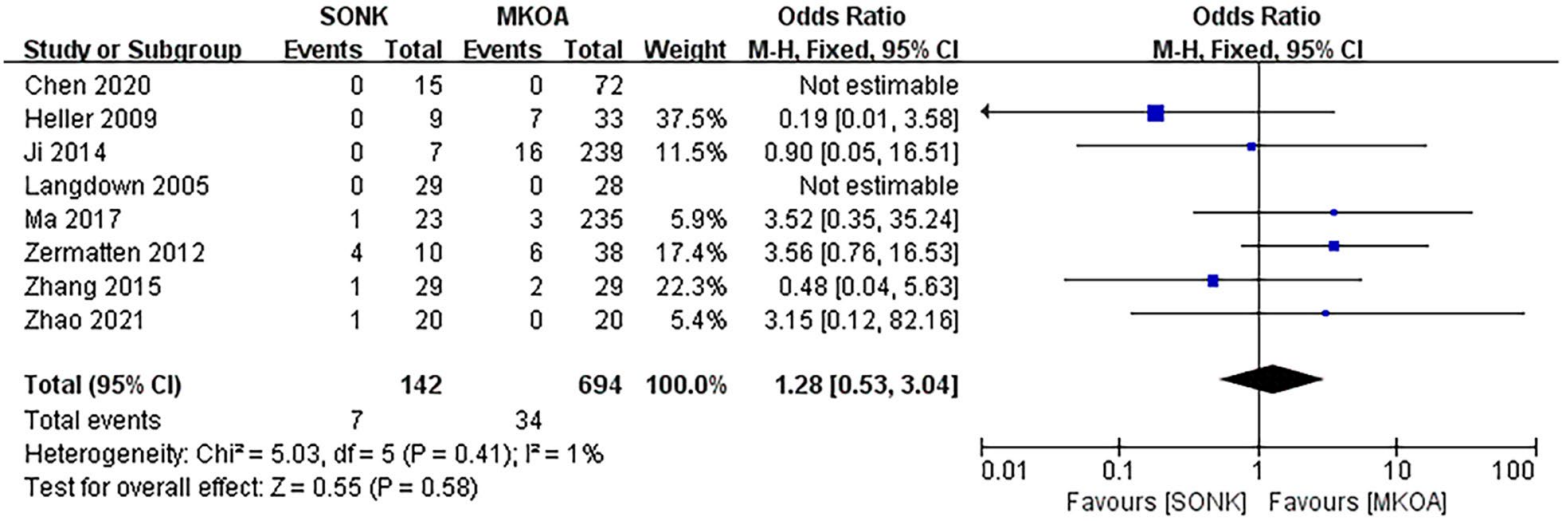
Comparison of postoperative revision rates between the SONK and MKOA groups

Eight papers [14–18, 20–22] compared revision rates at the final follow-up, totaling 836 knees, 142 in the SONK group and 694 in the MKOA group. There was little heterogeneity between the studies (*P *= 0.41, I^2^ = 1%), so a fixed-effects model was used. The results showed OR = 1.28, 95% CI (0.53, 3.04), *P *= 0.58, and the difference was not statistically significant (Fig. 8).

## Discussion

A total of six case‒control studies and three cohort studies were included in this meta-analysis to compare and analyse the clinical outcomes of OUKA in the treatment of patients with SONK and MKOA. The results showed that the postoperative functional evaluations, complications, and revision rates of OUKA in the treatment of SONK and MKOA were essentially the same, and the differences were not statistically significant.

There was no statistically significant difference between the SONK and MKOA groups in postoperative knee function scores and postoperative knee mobility, suggesting that similar efficacy can be achieved in functional evaluation between the two groups. In terms of the site of disease, MKOA occurs in the tibia in the anteromedial region and in the femur in the weight-bearing region, with varying degrees of contracture of the ligaments, whereas in SONK, the cartilage of the femur is most often exfoliated, and the ligaments are close to the normal anatomical level [2], so it is not surprising that the SONK group could achieve a similar level of function to the MKOA group after the removal of the exfoliated cartilage and the necrotic bone in the operation.

Comparison of the SONK and MKOA groups in terms of postoperative pain, rate of bearing dislocation, aseptic loosening, and postoperative progression of posterolateral arthritis did not show statistically significant differences, indicating that similar efficacy can be achieved in terms of postoperative complications. In terms of postoperative pain, the clinical outcomes of SONK and MKOA after OUKA were similar, but the preoperative subjective scores of the patients in SONK were worse than those in MKOA [17, 23], which indicated that the improvement in pain scores after OUKA surgery was greater in the SONK group than in the patients in the MKOA group and that OUKA could effectively alleviate the pain of the patients and that the difference in the degree of preoperative pain of the patients in the two groups may be associated with the pathogenesis of the two. Some current literature supports the association of SONK with subchondral bone insufficiency fracture in terms of pathology and imaging [14, 24], whereas OA is associated with a disruption of the balance between cartilage matrix repair and injury [25]. In terms of aseptic loosening, OUKA osteotomies are small, and areas of necrotic bone need to be completely removed. When intraoperative osteotomies do not remove the necrotic bone completely, the remaining necrotic bone needs to be scraped away, and depending on the size of the defect volume, the choice of using either bone cement or autogenous bone filler is made [3, 26]. When necrotic bone has not been completely removed, the prosthesis fitted on top of it may have an increased risk of loosening, whereas Shinichi conducted a study by treating 50 medial knee SONKs with OUKA and showed that regardless of the amount of necrotic bone shown in the respective cases, all patients had good or excellent results at the final follow-up; thus, Shinichi concluded that the limited amount of necrotic bone in the SONK had little impact on the rate of aseptic loosening of the UKA [27], which is similar to the clinical results of the MKOA, which is the same as the current article's findings. The authors concluded that the limited amount of necrotic bone in SONK, mostly small and medium-sized necrotic foci, is mostly amenable to OUKA, and when the necrotic foci are large, they need to be filled with autogenous bone grafts taken out during the surgery instead of bone cement; otherwise, it may lead to instability of the prosthesis [15, 17]. In terms of postoperative posterolateral arthritis progression, postoperative posterolateral osteoarthritis after OUKA usually occurs due to surgical overcorrection of the original inversion deformity [28], and the authors believe that how the result of the force line correction is not related to the difference in the type of disease between these two and the fact that the site of the onset of SONK is mostly confined to the unicompartmental compartment, with the lesion seldom spreading to the other compartments, so that there is no difference in the progression of the postoperative posterolateral arthritis between the two.

There was no statistically significant difference in revision rates between the SONK and MKOA groups. It was mentioned in some previous studies that the efficacy of UKA for SONK was poorer than that of MKOA [29], and this study used a noncemented fixation type of UKA, and the author believes that the reason may be that the installation of this type of prosthesis has a smaller amount of osteotomies, which does not completely remove the necrotic bone and has a poorer quality of subchondral bone, and that the failure to incorporate bone cement will result in unstable prosthesis placement, which in turn will lead to an increase in the loosening rate of the prosthesis and the revision rate, but a poorer bone quality has little effect on bone porosity and therefore does not affect the interlocking of bone and cement in cemented UKA [30], which in turn does not lead to an increased revision rate in the SONK group.

Our study has some limitations: (1) the number of included studies was small, the sample size was small, and all of them were retrospective; (2) because the quality of our study depends on the data from the original publications used in our meta-analysis, our study may not be able to avoid some of the potential bias and confounding effect issues of the included observational studies; (3) in some of the included studies, the OUKA used surgical technique and the grade of SONK lesions were not clearly defined for further evaluation; (4) the types of active spacer prostheses used for OUKA varied, and some types have been obsolete; (5) the mean follow-up time varied among the studies included in the meta-analysis; and (6) radiological outcomes could not be evaluated because of the limited data from the included studies.

## Conclusion

OUKA for the treatment of spontaneous osteonecrosis and medial compartment osteoarthritis of the knee can achieve similar satisfactory clinical results. OUKA is a valuable treatment option for SONK with good clinical outcomes and low failure rates. As the included literature is observational studies, the exact efficacy and safety evaluation awaits future randomised controlled trial studies with multicentre, large-scale and long-term follow-up. In addition, further studies are needed to specifically compare the outcomes of cementless UKA in SONK and MKOA.

## Data Availability

The data that support the findings of this study are available from the corresponding author.

## Abbreviations

OUKA: Oxford unicompartmental knee arthroplasty
SONK: Spontaneous osteonecrosis of the knee
MKOA: Medial knee osteoarthritis
UKA: Unicompartmental knee arthroplasty
PRISMA: Preferred Reporting Items for Systematic Reviews and Meta-Analyses
NOS: Newcastle‒Ottawa Scale
CI: Confidence interval
MD: Mean difference
OR: Odds ratio
